# A follow-up study of airway symptoms and lung function among residents and workers 5.5 years after an oil tank explosion

**DOI:** 10.1186/s12890-016-0357-3

**Published:** 2017-01-17

**Authors:** Jens-Tore Granslo, Magne Bråtveit, Bjørg Eli Hollund, Stein Håkon Låstad Lygre, Cecilie Svanes, Bente Elisabeth Moen

**Affiliations:** 1 Department of Occupational Medicine, Haukeland University Hospital, Bergen, Norway; 2 Department of Global Public Health and Primary Care, University of Bergen, Bergen, Norway; 3 Centre for International Health, University of Bergen, Bergen, Norway

**Keywords:** Airway symptoms, Environmental pollutants, Explosions, Lung function

## Abstract

**Background:**

Assess if people who lived or worked in an area polluted after an oil tank explosion had persistent respiratory health impairment as compared to a non-exposed population 5.5 years after the event.

**Methods:**

A follow-up study 5.5 years after the explosion, 330 persons aged 18–67 years, compared lung function, lung function decline and airway symptoms among exposed persons (residents <6 km from the accident site or working in the industrial harbour at the time of the explosion) with a non-exposed group (residence >20 km away). Also men in the exposed group who had participated in accident related tasks (firefighting or clean-up of pollution) were compared with men who did not. Data were analysed using Poisson regression, adjusted for smoking, occupational exposure, atopy and age.

**Results:**

Exposed men who had participated in accident related tasks had higher prevalence of lower airway symptoms after 5.5 years (*n* = 24 [73%]) than non-exposed men (28 [48%]), (adjusted relative risk 1.51 [95% confidence interval 1.07, 2.14]). Among men who participated in accident related tasks FEV_1_ decline was 48 mL per year, and 12 mL among men who did not (adjusted difference −34 mL per year [−67 mL, −1 mL]), and at follow-up FEV_1_/FVC ratio was 71.4 and 74.2% respectively, (adjusted difference −3.0% [−6.0, 0.0%]).

**Conclusion:**

Residents and workers had more airway symptoms and impaired lung function 5.5 years after an oil tank explosion, most significant for a group of men engaged in firefighting and clean-up of pollution after the accident. Public health authorities should be aware of long-term consequences after such accidents.

**Electronic supplementary material:**

The online version of this article (doi:10.1186/s12890-016-0357-3) contains supplementary material, which is available to authorized users.

## Background

On a morning in May 2007, an oil tank exploded and set fire to another tank with the same contents in a 1 km^2^ industrial harbour on a fiord in Western Norway.

The tanks contained airway-irritating chemicals such as hydrocarbons with a high content of sulphurous compounds, including mercaptans, hydrogen disulphide (H_2_S), and hydrochloride acid. The sulphur-rich coker gasoline had been added to the tanks from tank ships. Sweetening of the gasoline with caustic soda had resulted in a gasoline with lower sulphur content. The precipitated sludge on the bottom of the tanks had a high content of sulphurous compounds, and the tank self-ignited when hydrochloride acid was added to the tanks in order to dissolve the sludge.

Measurements of air pollution were scarce after the accident, but measurements performed 2–3 weeks after the accident demonstrated low levels of mercaptans. More detailed description of the incident and accidental exposure has been provided in earlier papers [1, 2].

About 1.5 years after the accident, a health examination was performed among adults who had been exposed to the pollution from the tanks, either because of local residence (less than 6 km from the accident site) or work place in the industrial harbour at the time of the explosion in May 2007. This was the baseline examination in a follow-up study, and a group of residents in the same municipalities as the industrial harbour area, not exposed to the pollution of the accident (residence more than 20 km from the accident place), was included as a non-exposed, control group. Local residents [1], and men with work place in the industrial harbour [2], had more upper and lower airway symptoms and signs of more obstructive lung function than the non-exposed, control group after 1.5 years.

Few follow-up studies have investigated the development of lung function and airway symptoms over a period of years after accidents with air pollution. One such study was performed after the World Trade Center (WTC) catastrophe in 2001, where fire-fighters and emergency service workers had considerable decline in lung function the first year after the incident, compared to the decline in lung function after the first year [3–5]. After the wreckage of the oil tanker ‘Prestige’, fishermen who took part in clean-up activity after the accident had significantly more lower airway symptoms, although not a larger decline in lung function, during the following years, as compared with subjects from a control group [6]. The exposure, accident circumstances, study design and start of health examination after the accident differed between these two incidents. Thus more evidence about the long-time respiratory health effect after accident-related air pollutions would be valuable.

The aim of this study was to examine whether a cohort of people who lived or worked in an area polluted after an oil tank explosion had persistent respiratory health impairment in the form of larger decline in lung function, lower lung function or more airway symptoms than a non-exposed population 5.5 years after the event.

## Methods

### Study design and population

A cohort was established 1.5 years after the explosion [1, 2]. Included as an exposed group were 18–67 year-old persons at the time of the first examination in 2008/2009 with residence <6 km from the accident site, or work place in the industrial harbour in May 2007. Included as a non-exposed, control group were residents 18–67 years old at the time of the same baseline examination with addresses >20 km (20–30 km) from the accident site, but within the same two municipalities as the exposed group. The individuals in the non-exposed group were age- and gender-matched with individuals in the exposed group. At baseline, names and addresses of residence were obtained from the National Population Register; lists with names of workers in the industrial harbour were obtained from their employers.

The cohort was examined twice: at baseline about 1.5 years (November 2008 - March 2009) and at follow-up about 5.5 years (November 2012 – March 2013) after the oil tank explosion, using a questionnaire and a health examination including spirometry and a blood sample. In total, 449 individuals participated at baseline in 2008/2009 (72% of the invited, 289 in the exposed group, 160 in the control group). At the follow-up in 2012/2013, 330 of these participated (53% of the invited, 218 in the exposed group and 112 controls).

Characteristics, airway symptoms and lung function among participants at baseline, comparing participants in this follow-up study with people who participated only at baseline (lost to follow-up), exposed and non-exposed individuals separately, are demonstrated in an on-line repository table (Additional file 1: Table S1 in on-line repository). FEV_1_, FVC and FEV_1_/FVC values are dependent on gender, age and height, and for these variables *P*-values are adjusted for gender, age and height when comparing follow-up and lost follow-up groups in the Table.

### Spirometer measurements

Both at baseline (2008/2009) and at follow-up (2012/2013), spirometry was performed with the same dry wedge spirometer, ‘Vitalograph Gold Standard plus’ (model 2160) in accordance with the ERS/ATS standardisation recommendation from 2005 [7], but accepting 200 mL repeatability [1, 2, 8]. Forced expiratory volume first second (FEV_1_) and forced vital capacity (FVC) was performed before and 15 min after inhaling 0.4 mg adrenergic beta-2-agonist salbutamol from a Discus inhalator [9, 10]. FEV_1_ and FVC in 2008/2009 and 2012/2013 were expressed in absolute values and as per cent predicted values according to a reference regression equation from a non-smoking, healthy, west-coast Norwegian population [11]. The annual change in FEV_1_ (∆FEV_1_), FVC (∆FVC) and FEV_1_/FVC ratio (∆FEV_1_/FVC) from the baseline examination in 2008/2009 to the follow-up examination in 2012/2013 was calculated. Spirometer pulmonary obstruction was defined as FEV_1_/FVC ratio <0.70, which is obstruction according to the GOLD criteria [12].

Body height and weight of participants were measured both in 2008/2009 and 2012/2013.

### Lower and upper airways symptoms

A similar questionnaire was used at baseline in 2008/2009 [1, 2], and at follow-up in 2012/2013. Questions of symptoms from lower airways were taken from the ATS-DLD-78A questionnaire [13, 14]. Answering “yes” to one or more of the nine questions: ‘Do you usually have morning cough?’; ‘daily cough?’; ‘cough at least 3 months a year?’; ‘cough with phlegm?’; ‘cough with phlegm at least 3 months a year?’; ‘dyspnoea walking on level ground?’; ‘dyspnoea walking uphill compared to others?’; ‘ever had episodes of wheezing?’ was defined as having lower airway symptoms.

Questions on current blocked nose, rhinorrhoea, irritated nose or sore throat included a five-point scale from none to severe (0–4) [1, 2, 15]. Answering 2 to 4 on the scale for one or more of these questions, defined upper airway symptoms.

### Exposure information and covariates

At baseline the following was assessed: ‘Did you take part in fire-fighting?’, or ‘did you take part in clean-up activities after the accident?’, and if yes, for how long [1, 2]. If individuals had taken part in fire-fighting or clean-up activities the first 6 months after the accident, they were defined as having taken part in accident-related tasks (ART). It was assumed that people in the exposed group who took part in accident related tasks had been more exposed to irritating airway pollution from the accident than people in the exposed group who did not take part in these tasks.

Assessment of smoking habit included years of daily smoking, present smoking (yes/no), previous/ex-smoking (yes/no), or never smoked (yes/no) [1, 2]. The questionnaire also included questions on infection in the preceding month (yes/no), having a cat or dog at home (yes/no), and having had moisture damage (yes/no) or carpeted floors at home (yes/no) [1, 2]. At follow-up in 2012/2013, the participants were also asked about educational level (only primary school, secondary/technical education up to 3 years after primary school (secondary/technical education), and four or more years after primary school (university/college). Current work status was assessed at baseline and follow-up (employed, sick leave or disability pension, retirement pension, student) [1, 2].

People in employment were asked to state their current occupation and industry using free text. In 2008/2009, occupations were coded in a standardised way [1, 2, 16], and each individual’s job was classified as either high occupationally exposed or low/not-exposed to biological dust, mineral dust or fumes/gases respectively, according to a general population job-exposure matrix (JEM) [1, 2, 17, 18]. The distribution of individuals’ classification in high or low/not occupationally exposed at follow-up in 2012/2013 was almost the same as was found at baseline in 2008/2009, and information from 2008 to 2009 was used as covariate in regression analyses.

### IgE measurements

A blood sample was taken at baseline in 2008/2009 and serum analyses were performed using Phadiatop® based on the Immuno-CAP-FEIA system (Phadia AB, Uppsala, Sweden). Subjects with positive Phadiatop® (specific IgE toward one or more of the following airway allergens: *Dermatophagoides pteronyssinus*, *Cladosporium herbarum*, cat, dog, horse, birch, timothy and mugwort) were defined as having atopy.

### Statistical analyses

The Pearson Chi-Square test or the Fisher exact test for small numbers was used to compare categorical characteristic variables between the exposed and the non-exposed group. Continuous variables were compared by independent sample *t*-test.

Multiple linear regression analyses were performed to compare annual mean change in FEV_1_ (∆FEV_1_) and FEV1/FVC ratio (∆(FEV_1_/FVC)) from baseline (2008/2009) to follow-up (2012/2013) between the exposed and non-exposed groups, while adjusting for smoking habit in 2008/2009 (present versus previous or never), occupational exposure in 2008/2009 (high versus low or none); atopy in 2008/2009 (having atopy versus not having atopy); age at the 2008/2009 examination (continuous scale) and height. The FEV_1_, FEV_1_% predicted, FVC, FVC % predicted and FEV_1_/FVC ratio in 2012/2013 was compared between the exposed and control groups in linear regression analyses, adjusting for smoking, occupational exposure, atopy, age and height, except for FEV_1_% predicted and FVC% predicted, where age and height were omitted. Only valid spirograms were used in the analyses [1, 2, 7, 8]. The risks of having different airway symptoms among exposed individuals as compared with the non-exposed group were measured as relative risks (RR) with 95% confidence intervals (95% CI). RRs were estimated in Poisson regression analysis with robust standard errors and also adjusted for smoking habits, occupational exposure, atopy and age.

The same analyses for lung function were also compared between the exposed men who took part in accident related tasks (fire-fighting or clean-up activities the first 6 months after the accident) (ART) with the exposed men who did not take part in these activities (No ART), but for respiratory symptoms the ART and the No ART groups were compared with the non-exposed group separately. Women were not included in these analyse since only one woman participated in the accident-related tasks.

Regression analyses with adjustments for possible confounding were not performed if the number of subjects with symptoms or obstruction were fewer than six in any of the groups.

The data were analyzed using SPSS 22.0 (Statistical Package for the Social Sciences, Statistical Products and Service Solutions, Inc., Chicago, IL). For analyses of relative risk (RR) of airway symptoms, analysis was also performed with STATA version 13.1 (Stat Statistics/Data Analysis, Software version 13.1, Stata Corp, College Station, TX) (for the log-binomial regression).

The study was approved by the Regional Committee for Medical Ethics of Western Norway (committee’s reference number: 20091) and Norwegian Social Science Data Services. Respondents had to sign a written consent declaration to participate in the study.

## Results

There were significantly more individuals with only primary school education in the exposed group than in the non-exposed group (Table 1). Other general characteristics and possible environmental respiratory health risks at home (having pets, moisture damage or carpets on floors: results not shown), did not differ significantly between the exposed and non-exposed groups (Table 1).

**Table 1:** General characteristics of 330 persons (exposed and non-exposed) who were investigated at baseline in 2008/2009 and at follow-up in 2012/2013, 18–67 years old at baseline

	Baseline (2008/2009)			Follow-up 2012/2013		
	Non-exposed	Exposed		Non-exposed	Exposed	
Total number	112	218		112	218	
	Mean (SD)	Mean (SD)	*p*	Mean (SD)	Mean (SD)	*p*
Age years	46 (12)	45 (12)	0.4	50 (12)	49 (12)	0.4^b^
Height m	1.73 (0.08)	1.75 (0.09)	0.2^b^			
Weight kg	80.1 (14.4)	81.9 (17.1)	0.4^b^	80.3 (13.7)	82.3 (16.8)	0.3^b^
BMI kg/m^2^	26.7 (4.3)	26.9 (5.1)	0.7^b^	26.8 (4.0)	26.9 (4.5)	0.8^b^
	*n* (%)	*n* (%)		*n* (%)	*n* (%)	
Gender						
Men	58 (52)	127 (58)	0.3^a^			
Women	54 (48)	91 (42)				
Social status						
In work	98 (87.5)	190 (87.2)	0.9 ^a^	89 (80.9)	181 (83.0)	0.6 ^a^
Retirement pension	2 (1.8)	4 (1.8)	1.0^a^	9 (8.1)	17 (7.8)	0.9 ^a^
Sick leave/rehabilitation	5 (4.5)	8 (3.7)	0.7^a^	4 (3.6)	13 (6.0)	0.4 ^a^
Disability pension	6 (5.4)	14 (6.4)	0.7^a^	7 (6.4)	7 (3.2)	0.2 ^a^
Student	8 (7.1)	12 (5.5)	0.6^a^	3 (2.7)	9 (4.1)	0.5 ^a^
Other	0 (0)	1 (0.5)		5 (4.5)	14 (6.4)	0.5 ^a^
Education						
Only primary school				4 (3.7)	30 (14.1)	0.02^a^
Secondary/technical education				56 (52.3)	112 (52.6)	1.0^a^
University/college				47 (43.9)	71 (33.3)	0.06^a^
Smoking habit						
Never	48 (45.3)	88 (41.3)	0.6^a^	49 (44.5)	83 (40.1)	0.3^a^
Ex- smoker	34 (32.1)	65 (30.5)		40 (36.4)	69 (33.3)	
Present	24 (22.6)	60 (28.2)		21 (19.1)	55 (26.6)	
Atopy (Phadiatop® positive)	25 (22.3)	49 (22.5)	0.8^a^			
High occupational exposure	30 (27.3)	57 (26.1)	0.8^a^			
Infection preceding month	35 (31.3)	60 (27.5)	0.5^a^	30 (26.8)	57 (26.1)	0.8^a^
Exposed group						
No ART	-	184 (84.4)				
ART	-	34 (15.6)				

*ART* Accident related tasks: firefighting or clean-up pollution after the accident

*SD* Standard deviation

Exposed group: Residence <6 km from accident place or work place in the industrial harbor in May 2007

^a^Pearson Chi-Square test

^b^Independent sampled *t*-test

The mean FEV_1_ decline (∆FEV_1_) per year during the 4-year follow-up appeared to be somewhat larger among exposed than non-exposed men, but this difference did not reach statistical significance in crude and adjusted analyses (Table 2). Changes in FEV_1_/FVC ratio (∆FEV_1_/FVC ratio) during follow-up did not differ significantly between exposed and non-exposed (Table 2).

**Table 2:** Change in FEV_1_ (∆FEV_1_) and FEV_1_/FVC ratio (∆FEV_1_/FVC ratio %) per year from baseline (2008/2009) to follow-up (2012/2013) in the exposed and non-exposed group, and among exposed men who did and did not take part in accident related tasks (ART)

	Number	∆FEV_1_ (mL/year)			*p*	∆FEV_1_/FVC ratio (%/year)			*p*
		crude mean (SEM)	Adjusted difference (95% CI)^a^			crude mean (SEM)	Adjusted difference 95% CI^a^		
Men									
Non-exposed l	49	−14 (10)	ref			−0.5 (0.1)	ref		
Exposed	105	−22 (4)	−14	(−40, 12)	0.3	−0.5 (0.1)	−0.1	(−0.5, 0.2)	0.5
Exposed men only									
No ART	78	−12 (8)	ref			−0.4 (0.1)	ref		
ART	27	−48 (14)	−34	(−67, −1)	0.04	−0.8 (0.2)	−0.4	(−0.9, 0.0)	0.05
Women									
Non-exposed	45	−15 (32)	ref			−0.5 (−0.2)	ref		
Exposed	74	−4 (6)	8	(−13, 28)	0.5	−0.4 (−0.1)	0.1	(−0.3, 0.6)	0.5

*ART* accident related tasks (fire-fighting or clean-up activities first 6 months)

*SEM* standard error of the mean

Adjusted difference: difference adjusted for smoking habit (in 2008), atopy, occupational exposure, age and height

^a^95% confidence interval

The sub-group of men within the exposed group who took part in accident-related tasks (fire-fighting or clean-up activities the first 6 months after the explosion) had a larger decline in FEV_1_ (48 mL/year) than men who did not take part in these activities (12 mL/year); this difference was statistically significant both in crude analyses (*p* = 0.03) and when adjusting for smoking habit, occupational exposure, atopy, age and height (*p* = 0.04) (Table 2).

Lung function before bronchodilation at follow-up was not significantly different in the exposed group compared to the non-exposed group (Table 3). Within the exposed group, men who took part in accident-related tasks (fire-fighting or clean-up activities the first 6 months) had a significantly lower FEV_1_/FVC ratio than men who did not participate in these tasks (*p* = 0.046) (Table 3).

**Table 3:** Lung function at follow-up in 2012/2013 in exposed and non-exposed group, and among exposed men who did and did not take part in accident related tasks (ART)

	Men				Women	
	Non-exposed	Exposed			Non-exposed	Exposed
		Total	No ART	ART		
Number	54	117	88	29	51	81
FEV_1_						
crude mean (SD)	3936 (794)	3890 (767)	3881 (735)	3917 (871)	2842 (485)	2935 (580)
Ad	Ref	−129	Ref	−46	Ref	53
95% confidence interval		(−331, 72)		(−225, 317)		(−99, 205)
*P*		0.3		0.8		0.4
FEV_1_% predicted^a^						
crude mean (SD)	93.5 (14.1)	91.0 (14.8)	91.8 (14.5)	88.8 (15.5)	96.0 (15.4)	95.4 (13.4)
Ad	Ref	−2.9	Ref	−1.4	Ref	1.1
95% confidence interval		(−7.6, 1.9)		(−7.7, 5.0)		(−4.0, 6.2)
*P*		0.2		0.7		0.7
FVC						
crude mean (SD)	5319 (846)	5286 (874)	5235 (849)	5440 (944)	3709 (579)	3812 (674)
Ad	Ref	−137	Ref	147	Ref	59
95% confidence interval		(−375, 100)		(−172, 466)		(−125, 243)
*P*		0.3		0.3		0.5
FVC% predicted^a^						
crude mean (SD)	104.0 (12.6)	101.7 (14.4)	101.6 (14.5)	101.8 (14.4)	102.5 (14.5)	101.9 (12.3)
Ad	Ref	−2.7	Ref	1.3	Ref	0.6
95% confidence interval		(−7.4, 2.0)		(−5.1, 7.6)		(−4.2, 5.5)
*P*		0.3		0.7		0.8
FEV_1_/FVC						
crude mean (SD)	73.8 (7.5)	73.6 (7.7)	74.2 (7.9)	71.4 (6.9)	76.6 (7.1)	76.9 (6.5)
Ad	Ref	−0.3	Ref	−3.0	Ref	0.2
95% confidence interval		(−2.5, 1.9)		(−6.0, 0.0)		(−2.1, 2.4)
*P*		0.9		0.046		0.8

*ART* Accident related tasks: Fire-fighting or clean-up activities first 6 months after accident

*SD* standard deviation

*Ad* adjusted difference: Difference adjusted for smoking habit (in 2008), atopy in 2008, occupational exposure, age and height

^a^FEV_1_% predicted and FVC% predicted related to a non-smoking, lung healthy population each compared with same gender and age 2012/2013

Airways obstruction before bronchodilation at follow-up defined as FEV_1_/FVC ratio <0.70, was slightly more common among men in the exposed group, and especially prevalent in the group with accident-related tasks (Table 4). The difference between the groups was not statistically significant in logistic regression analyses (results not shown).

**Table 4:** Airways obstruction at baseline (2008/2009) and at follow-up (2012/2013) in exposed and non-exposed group, men and women separately and among men who take part in accident related tasks

	Airways obstruction at baseline		Airways obstruction at follow-up	
	Before bronchodilatation	After bronchodilatation	Before bronchodilatation	After bronchodilatation
	*n* (%)	*n* (%)	*n* (%)	*n* (%)
Men				
Non-exposed	7 (13.5)	5 (9.4)	12 (22.2)	7 (13.2)
Exposed	26 (22.2)	16 (15.2)	32 (27.4)	24 (21.2)
Accident related tasks	4 (12.9)	3 (11.5)	9 (31.0)	5 (17.9)
Women				
Non-exposed	4 (8.3)	2 (4.1)	7 (13.7)	6 (11.8)
Exposed	8 (10.0)	5 (5.6)	11 (13.6)	7 (8.8)

Airways obstruction: FEV_1_/FVC ratio <0.70

Accident related tasks: fire-fighting or clean-up activities first 6 months after accident

Lower airway symptoms were significantly more common among exposed men than non-exposed, both at baseline in 2008/2009 with adjusted relative risk 1.38 (95% confidence interval 1.07–1.78) and at follow-up in 2012/2013 with adjusted relative risk 1.36 (95% confidence interval 1.00–1.84) (results not shown in Table). When stratifying exposed men in those who took part in accident related tasks (ART) and those who did not (No ART), ART group had significantly more prevalent lower airway symptoms than non-exposed men at follow-up, but No ART group was not significantly different from non-exposed men (Table 5). Among men in the exposed group who participated in accident-related tasks, 96% (*n* = 22) of those with lower airway symptoms in 2008/2009 still had symptoms in 2012/2013. In the sub-group of exposed men who did not take part in these tasks, 79% (*n* = 55) of those with symptoms at baseline still had symptoms, and in the non-exposed group the corresponding proportion was 71% (*n* = 22).

**Table 5:** Upper and lower airway symptoms at baseline (2008/2009) and at follow-up (2012/2013) in exposed and non-exposed group, and among exposed men who did and did not take part in accident related tasks (ART)

	Number	Lower airway symptoms								Upper airway symptoms							
		2008				2012				2008				2012			
		*n* (%)	RR	95% CI^a^	*p*	*n* (%)	RR	95% CI^a^	*p*	*n* (%)	RR	95% CI^a^	*p*	*n* (%)	RR	95% CI^a^	*p*
Men																	
Non-exposed	58	31 (53)	1			28 (48)	1			20 (35)	1			19 (33)	1		
No ART	94	70 (74)	1.41	(1.08, 1.83)	0.01	59 (63)	1.30	(0.95, 1.79)	0.1	52 (55)	1.57	(1.04, 2.35)	0.03	44 (47)	1.50	(0.96, 2.34)	0.07
ART	33	23 (70)	1.30	(0.94, 1.80)	0.1	24 (73)	1.51	(1.07, 2.14)	0.02	20 (61)	1.78	(1.13, 2.80)	0.01	16 (48)	1.51	(0.88, 2.57)	0.1
Women																	
Non-exposed	54	28 (48)	1			26 (48)	1			15 (28)	1			15 (28)	1		
Exposed	91	57 (63)	1.33	(0.95, 1.86)	0.1	50 (55)	1.08	(0.77, 1.51)	0.7	48 (53)	1.77	(1.10, 2.84)	0.02	46 (51)	1.80	(1.08, 2.99)	0.02

*ART* Accident related tasks: Fire-fighting or clean-up activities first 6 months after accident

*RR* Relative risk estimated in Poisson regression analysis with robust standard errors and adjusted for smoking habits, occupational exposure, atopy in 2008 and age

^a^95% confidence interval

Upper airway symptoms were significantly more common among the exposed at baseline, both for men and women: at follow-up this was only significant among women (Table 5).

In the questionnaires were also asked if the participants had asthma, and in the questionnaire at follow-up also if they had COPD (chronic obstructive pulmonary disease), but prevalence of reported asthma and COPD was not significantly different between exposed and non-exposed groups.

## Discussion

Residents and workers had more airway symptoms and impaired lung function 5.5 years after an oil tank explosion. Five and a half years after the explosion, men who took part in accident-related tasks (fire-fighting or clean-up activities) had accelerated FEV_1_ decline during the observation period, lower FEV_1_/FVC ratio at follow-up, and slightly more airways obstruction than exposed men who did not participate in these tasks. Compared to a non-exposed population, exposed women had a higher prevalence of upper airway symptoms, while exposed men who took part in accident-related tasks had higher prevalence of lower airway symptoms, 5.5 years after the accident.

In previously published findings from the baseline survey, we also observed an increase in airway symptoms and impairment in lung function 1.5 years after the accident [1, 2]. The findings from the follow-up study support the interpretation that exposure from the accident has led to chronic symptoms and lung function impairment.

In this study the yearly FEV_1_ decline over the 4-year follow-up period among all men who took part in the accident-related tasks was 48 mL a year, which was higher (54 mL) among ever-smokers in this group, (among ever-smoking men in the exposed group who did not take part in accident-related tasks, the FEV_1_ decline was only 11 mL a year). The FEV_1_ decline among non-smoking fire-fighters and emergency service workers (EMS) after the WTC catastrophe was considerably higher the first year after the accident (439 mL for fire-fighters and 267 mL for EMS workers), but the 7-year follow-up showed a yearly FEV_1_ fall of about 25 mL for fire-fighters and 40 mL for EMS workers after the first year [4]. We find a somewhat larger decline among men who took part in accident related tasks, between 1.5 and 5.5 years after the accident than was found among non-smoking firefighters between 1 and 7 years after the WTC catastrophe. However, in our study we had no information to assess potential decline during the first 1.5 years after the accident. The study design and accident related exposure was also different in the two studies.

In contrast, the follow-up study 2 and 6 years after the wreckage of the oil tanker ‘Prestige’, found that non-smoking fishermen who lived in the vicinity of and took part in the clean-up of the oil spill from the tanker had a lower yearly FEV_1_ decline during the follow-up period (about 10 mL a year) than a control group of non-smoking fishermen from the non-polluted part of the coast (about 30 mL a year) who did not take part in clean-up activities. The start and time span of the follow-up period after that accident was more comparable to our study than to the WTC study, but the accident and type of airway exposure differed from our study. However, in accordance with our findings, the Prestige study found a higher prevalence of lower respiratory symptoms in the exposed than the control group both at the start and at the end of the study [19].

The exposed group who did not participate in accident-related tasks had a comparable yearly FEV_1_ decline as the control group (12 and 14 mL a year). This is in agreement with the findings in the ‘Prestige’ study.

The expected physiological decline in FEV_1_ among never-smokers is about 25 mL a year after reaching expected maximal value at about 25–30 years of age [20, 21], but this varies between studies [22]. Persistent smoking is in many studies calculated to give an additional yearly decline of more than 10 mL, increasing with an increasing amount of cigarettes [22], and reported to be about 15 mL a year among heavy smokers [23, 24]. We included people from 18 years old at baseline and thus had participants who had not reached their expected maximal lung function, thus contributed to a relatively low estimate for yearly FEV_1_ decline. Some studies also indicate an additional yearly decline in FEV_1_ of 7–8 mL/year due to occupational exposure [23]. In Norwegian studies an additional yearly decline of about 6 mL was found in the smelting industry [23], about 11 mL among tunnel construction workers [25], and about 12 mL among aluminium plant workers [26]. Thus, the yearly decline in FEV_1_ among accident-related exposed men of 48 mL (54 mL among the ever-smokers) as observed among participants in the present study, is comparable to the decline observed among smokers in heavy industries with occupational exposure known to damage lung function. This supports a potential for an airways-damaging effect of the exposure from the presently described accident.

One strength of our study is that we used a non-exposed group which is comparable to the study group. The two groups came from the same area in Western part of Norway, and they were examined in the same, standardised way during the same seasonal period of the year, both at baseline in 2008/2009 and at follow-up in 2012/2013. Thus, the periods at baseline and at follow-up were comparable with respect to seasonal airway infections and exposure to common airway allergens. Furthermore, information about covariates which may be associated with temporary or long term respiratory health was collected, such as environmental exposure at home, infection during the preceding month, occupational exposure, personal habits and socioeconomic status such as educational level, work status, and IgE-atopy. We considered them as possible confounding factors by adjusting for them in regression analyses of airway symptoms and lung function.

Limitations in our study are lack of health information prior to the accident, and until the first examination about 1.5 years after the accident. Different respiratory health effects between the exposed and non-exposed groups may have been present before the accident. Thus, we cannot conclude with a causal relationship between exposure and health effects. Adjustment for other occupational exposure and smoking did not explain the findings: the number of never-smokers was too small to perform sensitivity analyses in this group.

Information is scarce about the level of environmental exposure from the smoke during the fire, and from irritating chemicals from polluted soil and water around the tanks in the aftermath of the accident. Moreover, we do not know the level of personal exposure to irritating chemicals on the airways, but it is assumed to have been highest for workers who took part in clean-up of the polluted material after the accident. Further stratification of this group based on the possible surrogates of exposure such as type of, and time spent on, clean-up activities, or the use of personal respiratory protecting equipment, could not be done due to the small sample size.

Environmental air pollution from production by local industry in the industrial harbour area may also contribute to respiratory health effects in the exposed group, since this harbour area has the highest concentration of industry in the municipality. In addition, across the fjord lies the largest oil refinery in Norway. However, there is no published information about measurements of air emissions from that refinery. Measurements of air pollution in the harbour area after the accident, first performed after 2–3 weeks, showed low exposure levels. Based on these measurements it is not likely that ordinary air pollution in the area is high. Except for the industrial harbour, there are no industrial emission sites in the two municipalities. Both municipalities are rural with very little road traffic, also just outside the 1 km^2^ industrial harbour. So, environmental air pollution is expected to be low in the two municipalities. However, no monitoring of environmental air pollution in the exposed (within 6 km from the accident site) and the non-exposed areas (more than 20 km away) have been performed to compare differences between the areas. Therefore, we cannot rule out that it may be some differences in environmental air pollution.

Occupational exposure may also contribute to respiratory health. Therefore each person in work was classified into an assumed high, low or non-exposed group based on where the individual’s job status was grouped according to a general population job-exposure matrix (JEM). The prevalence of high occupational exposure was the same in both the exposed and non-exposed groups. However, we have no information about individual occupational exposure, and therefore no information to verify whether our grouping into different occupational exposure levels gives a correct picture of the real individual occupational exposure. It may be a residual effect due to this uncertainty, but we do not know if this potential residual would have had a differential effect between the exposed and non-exposed groups, or, within the exposed group, between people who took part in accident-related tasks and those who did not.

## Conclusion

In conclusion, a follow-up study of respiratory health after an oil tank explosion showed persistent airways effects after 5.5 years, in the group of residents and workers. Increased prevalence of lower airway symptoms among exposed men, as found at baseline 1.5 years after the accident, persisted until follow-up 4 years later. This was most significant at follow-up in the group of men who had been engaged in fire-fighting and clean-up activities. This group had also accelerated lung function decline and more pronounced airways obstruction even after 5.5 years. It seems likely that air pollution from the accident has contributed to the observed impairment in respiratory health. Public health authorities should be aware of potential long-term respiratory health consequences after such accidents.

## Additional file

Additional file 1: Table S1. Comparing characteristics, airway symptoms and lung function at baseline (2008/2009) between participants in follow-up and participants only at baseline (lost follow-up), exposed and non-exposed separately. (DOCX 25 kb)

## Abbreviations

FEV_1_: Forced expiratory volume in one second.
FEV_1_% predicted: Forced expiratory volume in one second percent predicted.
FVC: Forced vital capacity.
GOLD: The Global Initiative for Chronic Obstructive Lung Disease
