# Supplementary material for: A follow-up study of airway symptoms and lung function among residents and workers 5.5 years after an oil tank explosion
Source: BMC Pulm Med. 2017 Jan 17;17:18. doi: 10.1186/s12890-016-0357-3 (PMC5240231; doi:10.1186/s12890-016-0357-3)
Supplement: Additional file 1: Table S1. — Comparing characteristics, airway symptoms and lung function at baseline (2008/2009) between participants in follow-up and participants only at baseline (lost follow-up), exposed and non-exposed separately. (DOCX 25 kb) [file 12890_2016_357_MOESM1_ESM.docx]

Table S1 on-line repositary . Comparing characteristics, airway symptoms and lung function at baseline (2008/2009) between participants in follow-up and participants only at baseline (lost follow-up), exposed and non-exposed separately.

|  |  |  | |  |  |  | |  |  |
| --- | --- | --- | --- | --- | --- | --- | --- | --- | --- |
|  |  | Non-exposed | |  |  | Exposed | |  |  |
|  |  | Lost follow-up | Follow-up |  |  | Lost follow-up | Follow-up |  |  |
|  |  |  |  |  |  |  |  |  |  |
| Number |  | 48 | 112 |  |  | 71 | 218 |  |  |
|  |  | Mean (SD) | Mean (SD) | P^a^ |  | Mean (SD) | Mean (SD) | P^a^ |  |
|  |  |  |  |  |  |  |  |  |  |
| Age | Years | 44 (13) | 46 (12) | 0.3 |  | 36 (12) | 45 (12) | <0.001 |  |
| Heigh | m | 1.75 (0.07) | 1.73 (0.08) | 0.3 |  | 1.74 (0.08) | 1.75 (0.09) | 1.0 |  |
| BMI | kg/m^2^ | 27.5 (5.0) | 26.7 (4.3) | 0.3 |  | 26.9 (6.0) | 27.0 (3.7) | 0.3 |  |
|  |  |  |  |  |  |  |  |  |  |
|  |  | n (%) | n (%) | P^b^ |  | n (%) | n (%) | P^b^ |  |
|  |  |  |  |  |  |  |  |  |  |
| **Gender** |  |  |  |  |  |  |  |  |  |
| Men |  | 27 (56.3) | 58 (51.8) | 0.7^c^ |  | 49 (69.0) | 127 (58.3) | 0.1^c^ |  |
| Women |  | 21 (43.7) | 54 (48.2) |  |  | 22 (31.0) | 91 (41.7) |  |  |
|  |  |  |  |  |  |  |  |  |  |
| **Social status** |  |  |  |  |  |  |  |  |  |
| In work |  | 39 (81.3) | 98 (87.5) | 0.3 |  | 61 (85.9) | 190 (87.2) | 0.8 |  |
| Retirement pension |  | 0 (0.0) | 2 (1.8) |  |  | 0 (0.0) | 4 (1.8) |  |  |
| Sick leave/rehabilitation |  | 2 (4.2) | 5 (4.5) | 0.9 |  | 6 (8.5) | 8 (3.7) | 0.1 |  |
| Disability pension |  | 5 (10.4) | 6 (5.4) | 0.2 |  | 2 (2.8) | 14 (6.4) | 0.2 |  |
| Student |  | 4 (8.3) | 8 (7.1) | 0.8 |  | 3 (4.2) | 12 (5.5) | 0.7 |  |
| Other |  | 0 (0.0) | 0 (0.0) |  |  | 2 (2.8) | 1 (0.5) | 0.1 |  |
|  |  |  |  |  |  |  |  |  |  |
| **Smoking habit** |  |  |  |  |  |  |  |  |  |
| Never |  | 31 (44.7) | 48 (45.3) |  |  | 21 (30.4) | 88 (41.3) |  |  |
| Ex-smoker |  | 18 (34.0) | 34 (32.1) | 1.0^d^ |  | 14 (20.3) | 65 (30.5) | 0.005^d^ |  |
| Present |  | 10 (21.3) | 24 (22.6) |  |  | 34 (49.3) | 60 (28.2) |  |  |
|  |  |  |  |  |  |  |  |  |  |
| Phadiotop positive |  | 15 (31.5) | 25 (22.3) | 0.2 |  | 22 (31.0)) | 49 (22.5) | 0.1 |  |
| High occupational exposure |  | 8 (17.0) | 31 (27.9) | 0.1 |  | 24 (33.8) | 57 (26.1) | 0.2 |  |
|  |  |  |  |  |  |  |  |  |  |
| **Exposed group** |  |  |  |  |  |  |  |  |  |
| No ART |  | 48 (100.0) | 112 (100.0) |  |  | 63 (88.7) | 184 (84.4) |  |  |
| ART |  | 0 (0.0) | 0 (0.0) |  |  | 8 (11.3) | 34 (15.6) | 0.4 |  |
|  |  |  |  |  |  |  |  |  |  |
| Lower airway symptoms |  | 25 (52.1) | 57 (50.9) | 0.9 |  | 45 (63.4) | 150 (68.8) | 0.4 |  |
| Upper airway symptoms |  | 17 (35.4) | 35 (31.1) | 0.6 |  | 34 (47.9) | 120 (55.0) | 0.3 |  |
|  |  |  |  |  |  |  |  |  |  |
| FEV_1_ reversibility ≥ 12% | % | 1 (2.1) | 2 (1.8) | 0.9 |  | 2 (2.8) | 6 (2.8) | 1.0 |  |
|  |  |  |  |  |  |  |  |  |  |
|  |  | Mean (SD) | Mean (SD) | P |  | Mean (SD) | Mean (SD) | P |  |
|  |  |  |  |  |  |  |  |  |  |
| FEV_1_ before bronchodilation | mL | 3526 (807) | 3464 (841) | 0.1 ^e^ |  | 3682 (824) | 3521 834) | 0.1^e^ |  |
| FEV_1_% predicted before bronchodilation | % | 91.3 (13.4) | 93.9 (13.5) | 0.3^a^ |  | 88.7 (13.4) | 91.1 (13.8) | 0.2^a^ |  |
| FVC before bronchodilation | mL | 4553 (955) | 4510 (1048) | 0.06^e^ |  | 4611 (940) | 4604 (1036) | 1.0^e^ |  |
| FVC% predicted before bronchodilation | % | 97.3 (11.6) | 100.3 (11.9) | 0.2^a^ |  | 93.1 (12.7) | 98.4 (12.7) | 0.005^a^ |  |
| FEV_1_/FVC ratio before bronchodilation | % | 77.5 (7.6) | 76.9 (6.7)^*^ | 0.2^e^ |  | 79.5 (6.6) | 76.6 (7.1) | 0.2^e^ |  |
|  |  |  |  |  |  |  |  |  |  |

BMI: body mass index.

SD: standard deviation

ART. Accident related tasks: firefighting or cleanup of pollution after the explosion in May 2007

FEV_1_ reversibility: (( FEV_1_  after bronchodilation with salbutamol - FEV_1_  before bronchodilation with salbutamol)/ FEV_1_  before bronchodilation with salbutamol) x 100.

^a^ Independent sampled *t*-test comparing follow-up with lost follow-up.

^b^ Pearson Chi-Square test comparing follow-up with lost follow-up.

^c^ Including both men and women gathered in analyses

^d^ Including smoking present, ex-smoker, never smoker gathered in analyses

^e^ Linear regression analyses comparing follow-up with lost-follow-up adjusting for gender, age and height.
